# Turbulent Drag Reduction by a Near Wall Surface Tension Active Interface

**DOI:** 10.1007/s10494-018-9918-2

**Published:** 2018-04-25

**Authors:** Somayeh Ahmadi, Alessio Roccon, Francesco Zonta, Alfredo Soldati

**Affiliations:** 1 Institute of Fluid Mechanics and Heat Transfer, TU Wien, Vienna, Austria; 2 Dipartimento Politecnico di Ingegneria e Architettura, Universitá di Udine, Udine, Italy

**Keywords:** Phase field method, Deformability, Interface, Wall bounded turbulence, Wall shear stress

## Abstract

In this work we study the turbulence modulation in a viscosity-stratified two-phase flow using Direct Numerical Simulation (DNS) of turbulence and the Phase Field Method (PFM) to simulate the interfacial phenomena. Specifically we consider the case of two immiscible fluid layers driven in a closed rectangular channel by an imposed mean pressure gradient. The present problem, which may mimic the behaviour of an oil flowing under a thin layer of different oil, thickness ratio *h*_2_/*h*_1_ = 9, is described by three main flow parameters: the shear Reynolds number *Re*_*τ*_ (which quantifies the importance of inertia compared to viscous effects), the Weber number *We* (which quantifies surface tension effects) and the viscosity ratio *λ* = *ν*_1_/*ν*_2_ between the two fluids. For this first study, the density ratio of the two fluid layers is the same (*ρ*_2_ = *ρ*_1_), we keep *Re*_*τ*_ and *We* constant, but we consider three different values for the viscosity ratio: *λ* = 1, *λ* = 0.875 and *λ* = 0.75. Compared to a single phase flow at the same shear Reynolds number (*Re*_*τ*_ = 100), in the two phase flow case we observe a decrease of the wall-shear stress and a strong turbulence modulation in particular in the proximity of the interface. Interestingly, we observe that the modulation of turbulence by the liquid-liquid interface extends up to the top wall (i.e. the closest to the interface) and produces local shear stress inversions and flow recirculation regions. The observed results depend primarily on the interface deformability and on the viscosity ratio between the two fluids (*λ*).

## Introduction

Fluid transportation inside pipelines and channels requires the application of an external pumping power to win the friction losses at the walls. When the adopted fluid is oil, the resulting pumping power is extremely large due to the large value of the oil viscosity. This has direct implications on the energy consumption which scales roughly with the third power of the transported flowrate. One possible strategy to limit the corresponding cost for fluid transportation is obtained by the injection of a low-viscosity fluid, which in most cases is water [1]. The effectiveness of this technique is due to the natural tendency of water to migrate towards the wall, in the high-shear region, so to lubricate the flow [2]. Since the pumping energy is spent to counterbalance the work done by the wall shear stress, viscous oil can be transported at the largely reduced cost of pumping water. Due to its importance in the petroleum industry and in the process and chemical engineering, the present flow configuration has been extensively studied and analyzed in the past [3, 3–6]. Literature in this field is vaste and old, dating back to the seminal industrial patents of [7] and [8]. An exhaustive literature review on the various aspects of the problem, together with an in-depth analysis of patents and solutions already existing is given for instance by [9] and [1].

Most of the literature on this subject is based on experimental observations with computational analyses on this type of turbulent drag reduction being a minor proportion. However, a number of theoretical works have investigated the changes occurring in the mechanism of transition to turbulence in pipes and channels with deformable wall. These studies used the linear stability analysis to examine the influence of a deformable boundary on the dynamics of Poiseuille flow [10, 11]. Linear stability analysis has been also explicitly employed to study the stability of a co-current flow of two immiscible fluids with different viscosities [12]. In the absence of surface tension, the flow is always unstable, with short-wavelengths instabilities arising at the interface between the two fluids [13]. When surface tension is taken into account, the resulting physics becomes more complex and requires the adoption of refined non-linear approaches to be analyzed [14]. We refer the interested reader to the recent review of [15] for an exhaustive overview on the onset and nature of instabilities in the broad field of viscosity stratified fluids. As discussed, most of the works on the motion of viscosity stratified liquids inside pipes and channels are based on experiments, and focus mainly on the quantification of global flow properties (flowrate and pressure drops) and on the qualitative characterization of the liquid-liquid interface structure. Obtaining a detailed time and space description of the entire flow field and of the liquid-liquid interface deformation is still an open issue for current experimental techniques. In this context, Direct Numerical Simulation (DNS) can be considered a useful tool to obtain the detailed evolution of the interface deformation together with an accurate description of the flow field in both phases. DNS has been successfully applied to the study of turbulence modulation over compliant walls or hyper-elastic layers [16, 17]. Recently, DNS has also proven accurate to analyze the complex time dependent three-dimensional dynamics of coupled gas-liquid turbulent flow [18–20], in particular to examine the physics of wave generation and the corresponding transfer rates of mass, momentum and energy across the interface. Compared to the case of gas-liquid flows, in which the number of DNS is constantly increasing, the case of liquid-liquid flows has attracted relatively less attention [14, 21, 22].

Motivated by this lack of detailed investigations on the problem, we started a systematic study with the object of examining the role of the interface dynamics in the process. In a previous paper [23] we have run a series of DNS using a Phase Field Method to characterize the viscosity-stratified liquid-liquid flow inside a turbulent flat channel. In that study we have been able to show that, compared to the case of a single phase flow driven by the same pressure gradient, the viscosity stratified liquid-liquid flow is characterized by a larger volume flowrate, due to the conversion of mean kinetic energy to potential energy of the deformed liquid-liquid interface. These results have demonstrated the presence of a certain degree of drag reduction even when the two-liquid layers have the same viscosity.

In the present study, we want to deepen and extend the analysis performed in our previous work characterizing more closely the interaction between the deformable liquid-liquid interface and the wall turbulence. We employ the same numerical methodology used in [23], which is based on pseudo-spectral DNS coupled with a Phase Field Method to track the dynamics of the liquid-liquid interface. Our simulations are run starting from a fully-developed velocity field of a single-phase turbulent channel flow. Later, the liquid-liquid interface is introduced so to obtain the desired configuration consisting of a thin less viscous layer flowing on top of a thicker and more viscous layer. The paper is built as follows. After a recap on the numerical methodology and on the effect of the liquid-liquid interface on the global properties of the flow (mean velocity, volume flowrate), we focus more closely on the interaction between the interface dynamics and the near wall turbulence. In particular, we find that the modulation of turbulence induced by the liquid-liquid interface is so important that wall shear stress inversions and local recirculation regions can be observed. These findings have been properly quantified and have been linked to the topology of the flow and of the deformed interface.

## Methodology

**Fig. 1:**
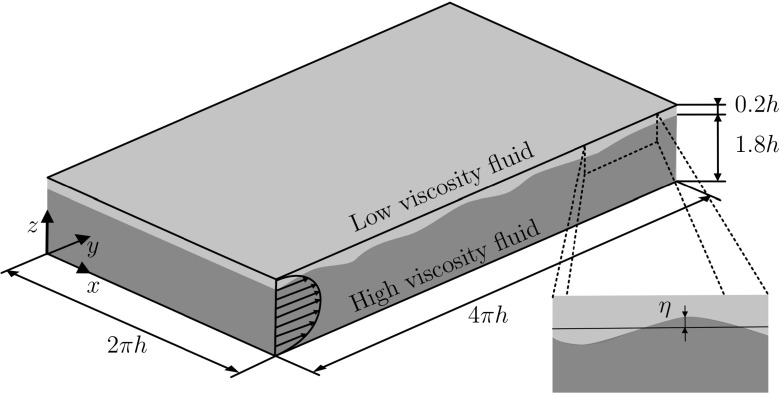
Sketch of the computational domain. A thin liquid layer with viscosity *ν*_1_ flows on top of a thicker liquid layer with viscosity *ν*_2_. The definition of the wave elevation *η* is also explicitly shown

With reference to the schematics shown in Fig. 1, we consider the case of two immiscible fluid layers flowing in a rectangular flat channel under the action of an imposed pressure gradient. The channel has dimensions *L*_*x*_ × *L*_*y*_ × *L*_*z*_ = 4*πh* × 2*πh* × 2*h* along the streamwise, spanwise and wall-normal directions, Fig. 1. The top part of the channel is occupied by a low-viscosity fluid layer (layer 1) characterized by a thickness *h*_1_, density *ρ*_1_ and viscosity *ν*_1_. The bottom part of the channel is occupied by a more viscous fluid layer (layer 2), characterized by thickness *h*_2_, density *ρ*_2_ and viscosity *ν*_2_. Specifically, we assume that the two fluids have the same density (*ρ*_1_ = *ρ*_2_) but different viscosity, so that a viscosity ratio *λ* = *ν*_1_/*ν*_2_ can be introduced. Note that in the present work we always set *λ* < 1 (i.e. the thin layer is always characterized by a smaller viscosity). The interface that separates the two phases is characterized by a constant value of the surface tension *σ* and is initially flat and located close to the top wall, such that *h*_1_ = 0.2*h* and *h*_2_ = 1.8*h*, i.e *h*_2_/*h*_1_ = 9.

The incompressible and Newtonian two phase flow system is modeled by a Phase Field Method (PFM). The basic idea of the Phase Field Method is the introduction of an order parameter *ϕ* that varies continuously over the interfacial layer and is uniform in the bulk phases (where *ϕ* = ± 1). Therefore, all the fluid properties can be rewritten as proportional to the order parameter to obtain a continuous change from one fluid to the other. The dimensionless equations describing the dynamics of the two-phase system read as [23–27]:

1 $$ \nabla \cdot \mathbf{u}= 0, $$

2 $$ {\partial\mathbf{u}\over{\partial t}} + \mathbf{u} \cdot \nabla \mathbf{u} = - \nabla p + \frac{1}{Re_{\tau}} \nabla \cdot [\kappa (\phi, \lambda)(\nabla \mathbf{u}+\nabla \mathbf{u^{T}})]+ \frac{3}{\sqrt{8}} \frac{Ch}{We} \nabla \cdot \tau_{c}, $$

3 $$ {\partial{\phi}\over{\partial t}} + \mathbf{u} \cdot \nabla \phi = \frac{1}{Pe} \nabla^{2}\mu_{\phi}, $$

4 $$ \mu_{\phi}=\phi^{3}-\phi -Ch^{2} \nabla^{2} \phi. $$

In the above system of equations, Eqs. 1–2 are the conservation of mass and momentum (Navier-Stokes) for the system, whereas Eqs. 3–4 are the transport equations of the order parameter *ϕ* (Cahn-Hilliard and chemical potential equations) and essentially describe the dynamical evolution of the liquid-liquid interface. In Eqs. 1–2–3, **u** = (*u*_*x*_, *u*_*y*_, *u*_*z*_) is the velocity vector, *p* is the pressure field, while the stress tensor *τ*_*c*_ = (|∇*ϕ*|**I** −∇*ϕ* ⊗∇*ϕ*) is used to impose the jump boundary conditions for the normal stress at the interface between the two fluids. The scalar field *κ*(*ϕ*, *λ*) is a dimensionless field introduced to account for the viscosity contrast between the two phases. In particular, we assume that the kinematic viscosity *ν* is proportional to the order parameter as [28, 29]

5 $$ \nu(\phi)= \nu_{2} \frac{1-\phi}{2} + \nu_{1} \frac{1+\phi}{2}. $$

After some algebra, viscosity can be rewritten as the sum of a uniform and a non uniform part [30–32]

6 $$ \nu(\phi)=\nu_{2} + \frac{(1+\phi)(\nu_{1}-\nu_{2})}{2}. $$

with the (dimensionless) expression of the non uniform part that finally becomes:

7 $$ \kappa(\phi,\lambda)= (\lambda-1)\frac{\phi+ 1}{2}. $$

The following dimensionless groups appear in Eqs. 2–3:

8 $$ Re_{\tau}=\frac{u_{\tau} h}{\nu_{2}}, \qquad We=\frac{\rho u_{\tau}^{2} h}{\sigma}, \qquad Ch=\frac{\xi}{h}, \qquad Pe=\frac{u_{\tau} h}{\mathcal{M} \beta}. $$

These groups have been obtained by turning the governing equations into dimensionless form using the half channel height *h* as reference length, the friction velocity $u_{\tau }=\sqrt {\tau _{w}/ \rho }$ (with *τ*_*w*_ the shear stress at the wall) as reference velocity and *ξ* as a characteristic length scale of the interface; $\mathcal {M}$ is the mobility while *β* is a parameter used to made the chemical potential dimensionless. From a physical viewpoint, the Reynolds number *Re*_*τ*_ is the ratio between the inertial and the viscous forces, defined using the viscosity of the thick layer *ν*_2_ as reference. The Weber number *We* is the ratio between the inertial and the surface tension forces. Specifically, small values of *We* identify a rigid interface, whereas large values of *We* represent an highly deformable interface. The Peclet number *Pe* is the ratio between diffusive and convective time scales of the interface, while the Cahn number *Ch* is a measure of its dimensionless thickness.

The governing equations are solved using a pseudo-spectral method that transforms field variables into wavenumber space through the use of a Fourier representation along the streamwise and spanwise (periodic) directions and a Chebyshev representation along the inhomogeneous wall-normal direction. Equation 2 is recasted in a velocity-vorticity formulation, whereas for Eq. 3 an operator splitting is applied. In particular, Eq. 2 is replaced by a 4^*th*^ order equation for the wall-normal component of the velocity *u*_*z*_ and a 2^*nd*^ order equation for the wall-normal component of the vorticity *ω*_*z*_ [33], while Eq. 3 is splitted in two 2^*nd*^ order equations [26].

Time advancement is achieved through an IMEX scheme in which the linear terms are discretized by an implicit Crank-Nicolson scheme while the non-linear terms are discretized by an explicit Adams-Bashforth scheme.

At the channel walls a no slip condition is enforced for the velocity field:

9 $$ \mathbf{u}(\pm h)= 0, $$

A zero-flux boundary condition is enforced for both the order parameter *ϕ* and the chemical potential *μ*_*ϕ*_. This is formally equivalent to impose the following conditions:

10 $$ \frac{\partial \phi}{\partial z}(\pm h)= 0, \qquad \frac{\partial^{3} \phi}{\partial z^{3}}(\pm h)= 0, $$

whose application leads also to the conservation of the order parameter *ϕ* over time [25]. We consider four different cases: a single phase flow and three multiphase cases. All simulations are run at a reference Reynolds number *Re*_*τ*_ = 100 (defined based on the physical properties of layer 2, i.e. the thicker one). Simulation labelled *SP* is the reference simulation run for a single phase flow. In simulations *S*1 − *S*3, we consider a two-phase flow where the thin layer has a reduced viscosity (*λ* = 1 for *S*1, *λ* = 0.875 for *S*2 and *λ* = 0.75 for *S*3). The value of the surface tension characterizing the liquid-liquid interface is set through the Weber number, here kept constant, *We* = 0.1. To solve the complex turbulence-interface interactions, a minimum of 3 points must be prescribed across the interface thickness. This is achieved by setting the value of the Cahn number to *Ch* = 0.02. Once *Ch* is fixed, the Peclet number can be obtained as *Pe* ∝ *α*/*Ch* = 150 [34]. An overview of all the parameters used in the simulations is reported in Table 1. The domain is discretized with *N*_*x*_ × *N*_*y*_ × *Nz* = 512 × 256 × 257 grid points along the streamwise, spanwise and wall-normal directions respectively. Grid spacing, expressed in wall units, is ${\Delta } x^{+}={\Delta } y^{+}= 2.464~w.u., {\Delta } z^{+}_{min}= 0.014~w.u.$ (wall) and ${\Delta } z^{+}_{max}= 1.222~w.u.$ (center). In a fully developed turbulent channel flow at *Re*_*τ*_ = 100, the smallest value of the Kolmogorov length scale (found at the wall) is $\eta _{k}^{+}= 1.676~w.u.$ This value could allow for a coarser grid. However, this high resolution is required to reduce at most the value of *Ch* so to capture at best the interface dynamics. In the following, the quantities are expressed in wall units based on the fluid properties of the single phase flow:

11 $$ \mathbf{x}^{+}=Re_{\tau} \mathbf{x} \qquad t^{+}=Re_{\tau} t \qquad \mathbf{u}^{+}= \mathbf{u} \qquad \phi^{+}=\phi $$

where **u**, **x**, *t* and *ϕ* are the variables obtained from the solution of Eqs. 1–4.

**Table 1:** Overview of the parameters used for the four different simulations performed

Sim.	*R* *e*_*τ*_	*λ*	*W* *e*	*C* *h*	*P* *e*
*SP*	100	–	–	–	–
*S*1	100	1.000	0.1	0.02	150
*S*2	100	0.875	0.1	0.02	150
*S*3	100	0.750	0.1	0.02	150

Simulation labelled *SP* refers to a single phase flow, while *S*1-*S*2 and *S*3 are two-phase flow simulations of viscosity stratified flow (at different *λ*)

## Results

**Fig. 2:**
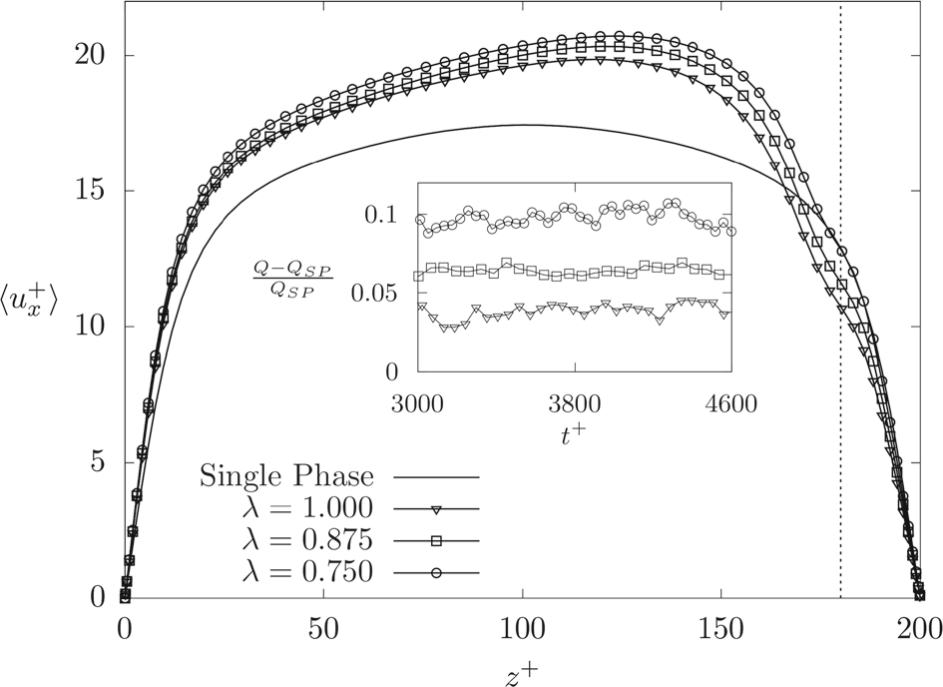
Wall-normal behavior of the mean fluid streamwise velocity $\langle u^{+}_{x} \rangle $ for the different simulated cases. Symbols refer to the viscosity stratified flows at different *λ*, whereas solid line refers to the single-phase case. The time behavior of the normalized deviation of the volume flow rate *Q* − *Q*_*SP*_ normalized by the value computed for the single phase flow *Q*_*SP*_ is shown in the inset. Adapted from [23]

We start all our simulations from the same initial condition, consisting of a single phase fully developed turbulent channel flow at *Re*_*τ*_ = 100. Then, we set the order parameter *ϕ* so to obtain the targeted setup characterized by a thin less viscous fluid layer flowing on top of a thicker and more viscous fluid layer. We begin our discussion on the dynamics of the two-phase liquid-liquid flow by looking at the wall-normal behavior of the mean streamwise velocity $\langle u^{+}_{x}\rangle $ for all the simulated cases, as shown in Fig. 2. Angular brackets 〈⋅〉 denote averaging in time and over the *x*^+^ − *y*^+^ homogeneous directions. Note that statistics are obtained upon averaging over a time window Δ*t*^+^ = 2000 after a statistically steady state is reached. We clearly notice that, regardless of the value of the viscosity ratio *λ*, the presence of the liquid-liquid interface (whose initial position is indicated by the dotted line at *z*^+^ = 180 in Fig. 2) influences the mean flow velocity. This influence is particularly pronounced near the interface, but it is also important in the core of the channel, where a remarkable increase of the mean velocity is observed. Due to the increase of the mean flow velocity, we expect a corresponding increase of the volume flow rate of the thicker and more viscous layer, *Q*. This is indeed explicitly shown in the inset of Fig. 2, where we plot the time behavior of the normalized quantity (*Q* − *Q*_*SP*_)/*Q*_*SP*_ (where *Q*_*SP*_ is the volume flow rate for the single phase flow) as a function of *λ*. It is apparent that even for the case in which the two layers have the same viscosity (*λ* = 1, Simulation *S*1), there is a mild, though not negligible (≃ 4*%*) increase of the volume flow rate. For decreasing *λ*, the volume flow rate further increases up to ≃ 10*%* for *λ* = 0.75. Since our simulations are run imposing the same pressure gradient, an increase of the volume flow rate likely indicates a decrease of the overall friction factor. Specifically, we find an increase of the shear stress at the bottom wall (from + 26.7*%* for *λ* = 1 up to + 31.7*%* for *λ* = 0.75), associated to an equivalent decrease of the shear stress at the top wall. Due to the increase of the overall bulk velocity, we observe a corresponding decrease of the friction factor *C*_*f*_ (normalized by the value observed for the single phase flow, $C_{f}^{SP}$) from $C_{f}/C_{f}^{SP}= 0.856$ for *λ* = 1, down to $C_{f}/C_{f}^{SP}= 0.747$ for *λ* = 0.75 [23]. This observation supports the idea that the turbulent drag reduction observed here is not only due to the reduced viscosity, but it is indeed due to the presence of a liquid-liquid interface that converts the Turbulent Kinetic Energy (TKE) produced close to the wall into potential energy of interface deformation.

**Fig. 3:**
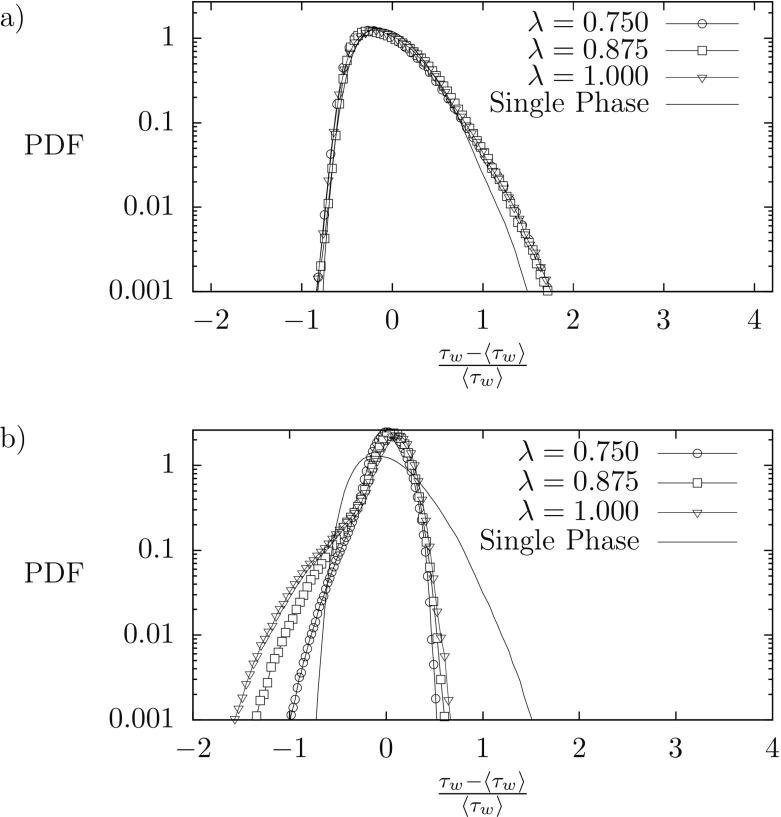
Probability Density Function (PDF) of the normalised wall shear stress fluctuation $\tau ^{\prime }_{w}=(\tau _{w} - \langle \tau _{w} \rangle ) / \langle \tau _{w} \rangle $ for all the simulated cases. **a** refers at the bottom wall (*z*^+^ = 0) while the **b** refers at the top wall (*z*^+^ = 200). The profile for the two phase cases (resp. single phase case) are shown with symbols (resp. solid line)

To characterize better the influence of the liquid-liquid interface on the near wall activity, we compute the Probability Density Function (PDF) of the wall shear stress at the top and bottom wall. In particular, we consider the normalized deviation of the wall shear stress with respect to the mean value at the corresponding wall, i.e. $\tau ^{\prime }_{w}=(\tau _{w}-\langle \tau _{w} \rangle )/\langle \tau _{w} \rangle $. Results, which are plot in semi-logarithmic scale, are shown in Fig. 3. Symbols refer to the two-phase flow simulations at different *λ*, whereas the solid line indicates the single phase case. At the bottom wall, Fig. 3a, only slightly influenced by the liquid-liquid interface, the behavior of the PDF$(\tau ^{\prime }_{w})$ of the multiphase flow cases (symbols) is similar to that of the single phase case (line). For all presented cases, PDF$(\tau ^{\prime }_{w})$ is not symmetric and is positively skewed, i.e it has a longer positive tail compared to the negative one. This indicates that large positive fluctuations occur more frequently than large negative fluctuations. The general shape of the PDF is consistent with that reported in previous literature studies [35, 36]. Deviations due to the presence of the liquid-liquid interface are visible only as a slight increase of the PDF for $\tau ^{\prime }_{w}>1$, a circumstance that can be ultimately linked to the slight increase of the mean shear stress at that wall. At the top wall, the situation is sharply different; only for the single-phase case the PDF$(\tau ^{\prime }_{w})$ is identical to that observed at the bottom wall (there is no statistical difference between the two walls for the single phase case). For all the other cases, the PDF shape is completely different. In particular, we notice that for decreasing *λ* the PDF $(\tau ^{\prime }_{w})$ becomes taller and narrows around the most probable value $\tau ^{\prime }_{w}= 0$. This indicates that wall shear stress fluctuations are largely reduced by the presence of the liquid-liquid interface, which indeed weakens the turbulent generation cycle and reduces the wall shear stress fluctuations. We interestingly notice that, while for the single phase case the PDF$(\tau ^{\prime }_{w})$ is positively skewed, for the multiphase cases it is negatively skewed. Yet, we observe the existence of a remarkable difference among the PDF profiles of the multiphase cases (symbols) for $\tau ^{\prime }_{w} \le -0.3$. In particular, the probability of observing large negative values of $\tau ^{\prime }_{w}$ ($\tau _{w}^{\prime }<-1$) increases for increasing *λ*. This is extremely important, because the appearance of $\tau _{w}^{\prime }<-1$ events indicates the presence of regions characterized by a local value of the shear stress *τ*_*w*_ that changes sign compared to the mean value measured at the same wall. Such shear inversions are a clear footprint of local flow recirculation patterns. Note that the onset and persistency of recirculation patterns is specifically linked to the presence of a liquid-liquid interface (characterized by a finite value of the surface tension) that interacts with the near wall turbulence modifying its dynamics. By contrast, these recirculation patterns depend only slightly on the value of the liquid viscosity, with a larger effect observed for *λ* = 1 (i.e. recirculation patterns have a reduced strength when the viscosity of the thin layer is reduced, i.e. when *λ* < 1). This observation is consistent with the possible turbulence reactivation by the reduced viscosity of the thin liquid layer. A decrease of fluid viscosity (decrease of *λ*) indeed induces an increase of the local Reynolds number [37].

**Fig. 4:**
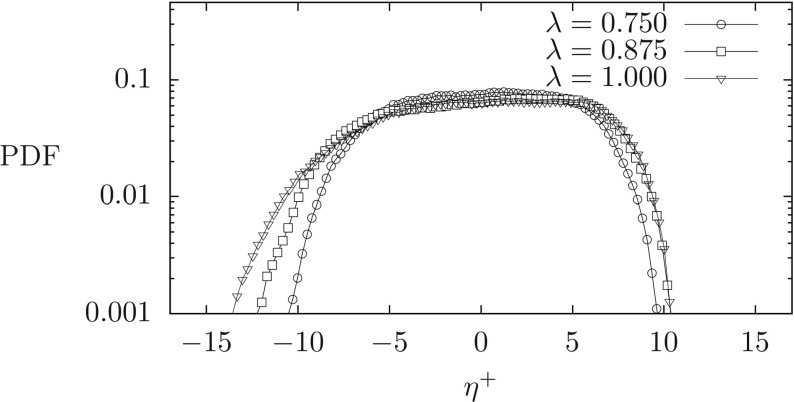
Probability Density Function (PDF) of the interface elevation *η*^+^ for all the simulated cases (different values of *λ*)

To understand the reason why we observe such a peculiar structure of the wall shear stress at the top wall, we now focus on the behavior of the interface dynamics. Our intuition is that the deformation of the liquid-liquid interface can induce a large turbulence modulation characterized also by the appearance of regions of local flow recirculation. To this aim, we calculate the PDF of the liquid-liquid interface elevation *η*^+^, expressed in wall units and computed as the difference between the actual position of the deformed interface and the initial position (see also the inset of Fig. 1 for the definition of *η*). Results are shown in Fig. 4. Positive values of *η*^+^ indicate an interface crest, whereas negative values of *η*^+^ indicate an interface throat. We observe that the PDF(*η*^+^) has a characteristic shape, regardless of the value of *λ*. The most probable values of the interface elevation range between − 5 < *η*^+^ < 5, with maximum at *η*^+^ ≃ 10 and minimum at *η*^+^ ≃− 15. This means that the interface deforms but it does not reach the wall (for which it should be *η*^+^ ≃ 20) and it does not break. We interestingly note that decreasing *λ* increases the probability of having − 5 < *η*^+^ < + 5 while reducing the probability of having large positive (*η*^+^ > 5) and negative (*η*^+^ < − 5) events. This reflects the observation that for *λ* = 1 the interface is smoother, yet characterized by higher crests and deeper throats.

For this reason, and without loss of generality, in the following we will focus on the case *λ* = 1, because the modulation of wall turbulence (induced by the liquid-liquid interface dynamics) we wish to discuss is emphasized at this value of *λ*.

**Fig. 5:**
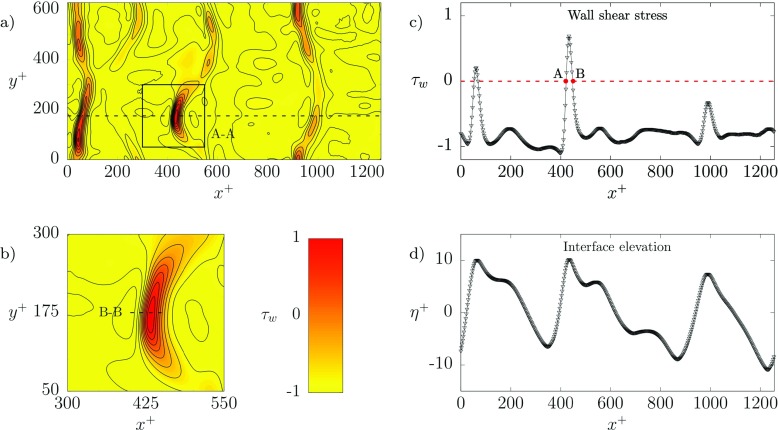
**a** Instantaneous wall shear stress distribution *τ*_*w*_ at the top wall (*z*^+^ = 200) computed at *t*^+^ = 3650 for the simulation with *λ* = 1 (simulation S1). Red regions identify positive areas of shear stress, i.e. opposite to the direction of the mean stress at that wall (〈*τ*_*w*_〉 < 0). **b** detailed inspection of the shear stress distribution in the square region A-A identified in panel **a**). **c-d** Streamwise distribution of the shear stress (panel c) and of the interface elevation (**d**) computed at *y*^+^ = 175

In Fig. 5 we focus on the instantaneous distribution of the shear stress at the top wall (*z*^+^ = 200) and the relative interface shape. We remark that the mean shear is negative at this wall, i.e. 〈*τ*_*w*_〉 < 0. In Fig. 5a-b, yellow regions indicate regions of negative *τ*_*w*_ (i.e. aligned with the mean shear) whereas red regions indicate regions of positive *τ*_*w*_ (i.e. at odds with the mean shear). The presence of regions in which *τ*_*w*_ changes sign compared to its mean value indicates the presence of a separation point in the boundary layer and a corresponding flow recirculation region. One of such recirculation regions is shown more closely in Fig. 5b and corresponds to the square highlighted in Fig. 5a and labelled A-A. As mentioned above, recirculation regions associated to the boundary layer separation are intimately linked to the dynamics of the underlying liquid-liquid interface. To draw this link, in Fig. 5c-d we present a vis-a-vis comparison between *τ*_*w*_ and *η*^+^ measured along the dashed line shown in Fig. 5a (and located at *y*^+^ = 175). Fig. 5c refers to the behavior of *τ*_*w*_, whereas Fig. 5d refers to the behavior of *η*^+^. In Fig. 5c the threshold *τ*_*w*_ = 0 is explicitly shown using a red dashed line. The correlation between *τ*_*w*_ and *η*^+^ is apparent. When *η*^+^ develops a peak, the corresponding value of *τ*_*w*_ trespasses the threshold line *τ*_*w*_ = 0 and favors the appearance of a recirculation region. This happens twice at the selected time and *y*^+^ position, namely at *x*^+^ ≃ 50 and *x*^+^ ≃ 420. A further extreme event for *η*^+^ occurs at *x*^+^ ≃ 1000, but in this case the interface elevation is not sufficiently large to induce the boundary layer separation.

**Fig. 6:**
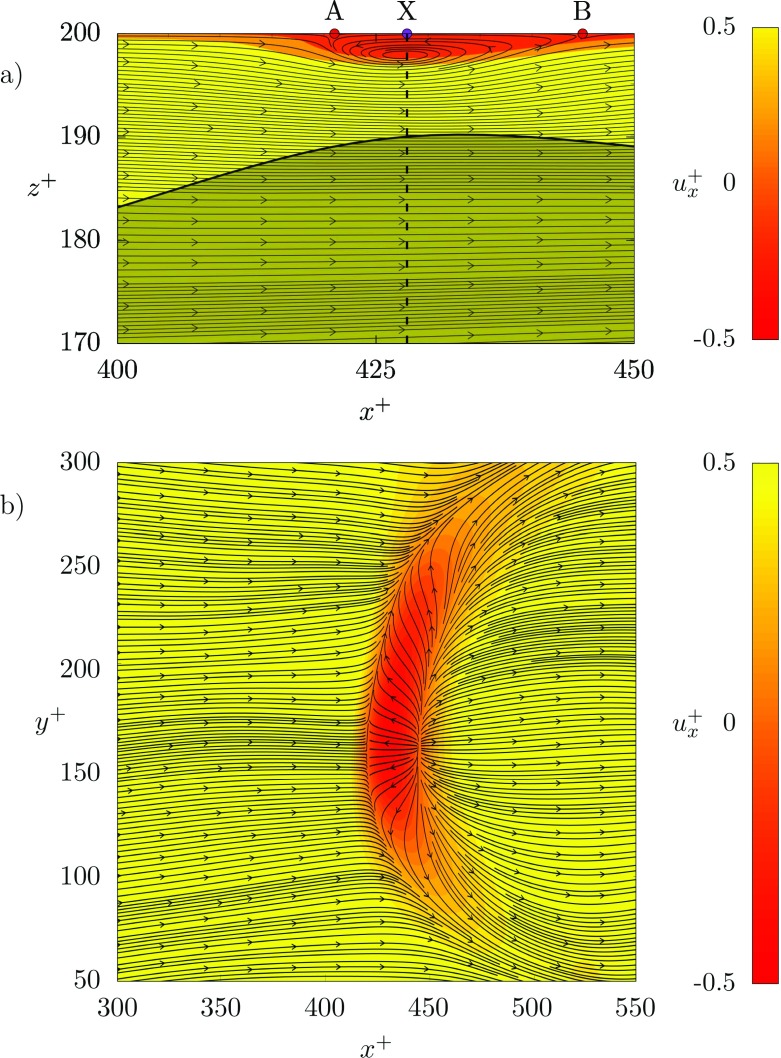
**a** Instantaneous flow streamlines corresponding to the region labelled B-B in Fig. 5. The *x*^+^-*z*^+^ plane is located at *x*^+^ = 175. The interface is indicated by a solid black line. **b** Instantaneous flow streamlines corresponding to the region labelled A-A in Fig. 5a. The *x*^+^-*y*^+^ plane is located close to the top wall, *z*^+^ = 199.5. In the two panels contour maps of the streamwise velocity are also shown: red regions correspond to regions of negative streamwise velocity (flow recirculation) whereas yellow regions correspond to regions of positive streamwise velocity

To understand more closely the reason why the boundary layer separates when the liquid-liquid interface develops a peak, we consider more closely the dynamics inside the region labelled A-A in Fig. 5. Results are shown in Fig. 6, with the recirculation region being explicitly visualized through the use of instantaneous flow streamlines. Fig. 6a refers to an *x*^+^ − *z*^+^ plane taken at a spanwise location *y*^+^ = 175 and ranging between 400 < *x*^+^ < 450 and 170 < *z*^+^ < 200. The points A and B correspond to those highlighted in Fig. 5c. Together with the flow streamlines, we also plot the value of the streamwise velocity (background color map) and of the interface elevation (solid thick black line). By looking at this picture we notice that the maximum interface elevation occurs at *x*^+^ ≃ 425, whereas the recirculation region ranges between 420 < *x*^+^ < 445 and has a thickness of roughly 5 wall units in the wall-normal direction. To further investigate on the structure of the recirculation region, a top view is reported in Fig. 6b. This picture shows the instantaneous flow streamline on a *x*^+^ − *y*^+^ plane between 300 < *x*^+^ < 550 and 50 < *y*^+^ < 300 and taken at *z*^+^ = 199.5 (i.e. very close to the top wall). We observe that the recirculation region has a crescent-shaped structure delimited by two curved stagnation lines (which include points A and B when *y*^+^ = 175). To explain such a peculiar structure, we considered the two dimensional divergence, ∇_2*D*_ [38]:

12 $$ \nabla_{2D}= \frac{\partial u^{+}_{x}}{\partial x^{+}}+\frac{\partial u^{+}_{y}}{\partial y^{+}}=-\frac{\partial u^{+}_{z}}{\partial z^{+}}. $$

**Fig. 7:**
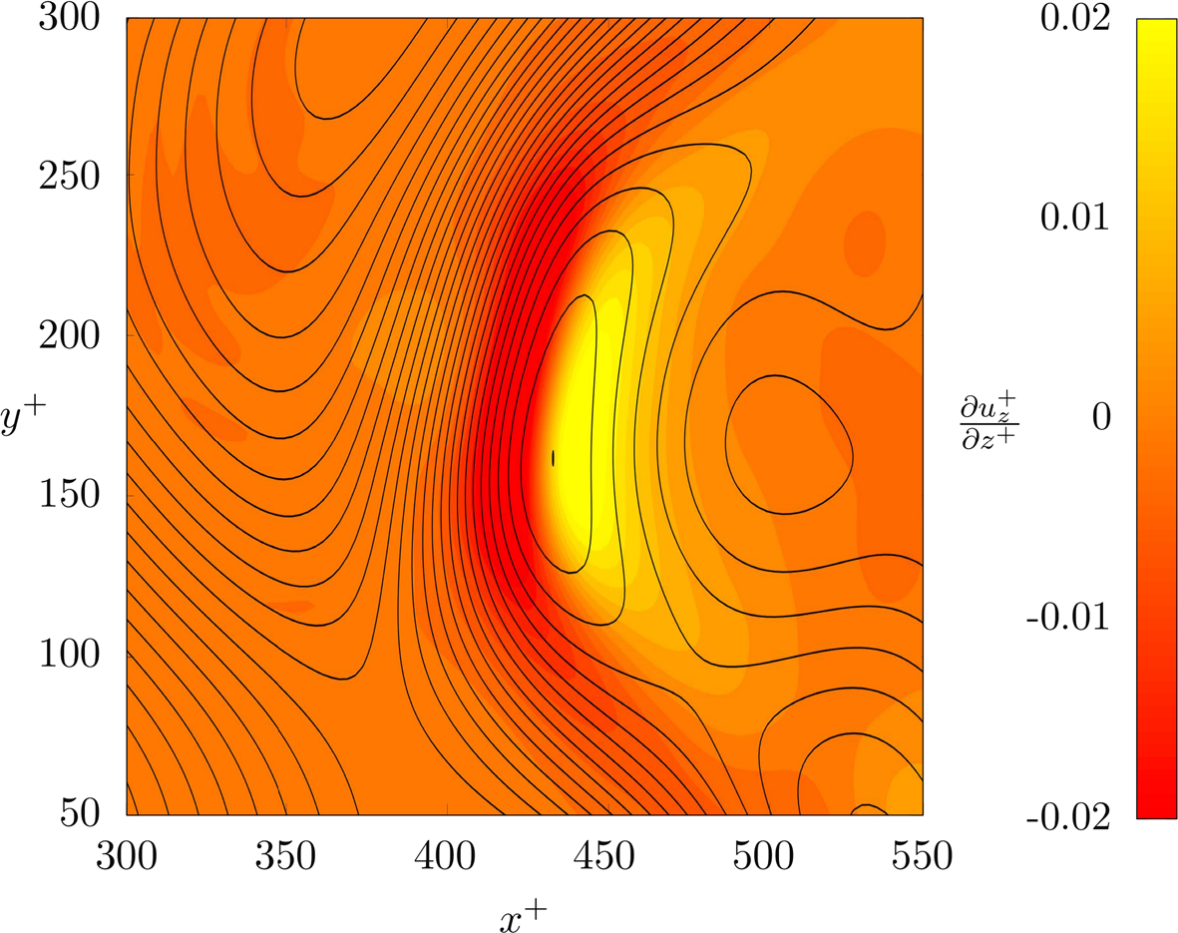
Contour plot of the two dimensional flow divergence, $\nabla _{2D}= -\frac {\partial u^{+}_{z}}{\partial z^{+}}$ for the square area labelled A-A indicated in Fig. 5. The horizontal slice is located at *z*^+^ = 199.5. Red regions identify upwellings while yellow regions identify downwellings. Contour lines (solid black lines) of the interface elevation *η*^+^ are also shown for comparison

We computed the two dimensional divergence in a *x*^+^ − *y*^+^ close to the top wall, at *z*^+^ = 199.5, Fig. 7. Upwards motions of fluids impinging on the wall (i.e. velocity sources usually called upwellings) are characterized by positive values of ∇_2*D*_, whereas downwards motions of fluid leaving the wall (i.e. velocity sinks usually called downwellings) are characterized by negative values of ∇_2*D*_. Results clearly indicate that there is a strong downwelling region (420 < *x*^+^ < 440) that slightly precedes a strong upwelling region (440 < *x*^+^ < 460). Based on our results, we envision the following mechanism. When the interface develops a peak, (for instance at *x*^+^ = 425, see Fig. 7) a lump of fluid in the thin liquid layer is pushed towards the top wall. Since it has a small (but not negligible) streamwise velocity, it impact the wall producing a stagnation point at *x*^+^ ≃ 445. Upon impaction with the top wall, the lump of fluid is split into two branches: one branch moves downstream (towards larger *x*^+^) while one branch moves upstream (towards smaller *x*^+^) and produces a recirculation region. However, due to the small inertia of this second branch and to the presence of the downstream flow, the recirculation region extends up to *x*^+^ = 420 and cannot move further upstream. When the incoming stream reaches the recirculation region, it is forced to move sideways (blockage effect of the recirculation region) generating the peculiar crescent shape.

**Fig. 8:**
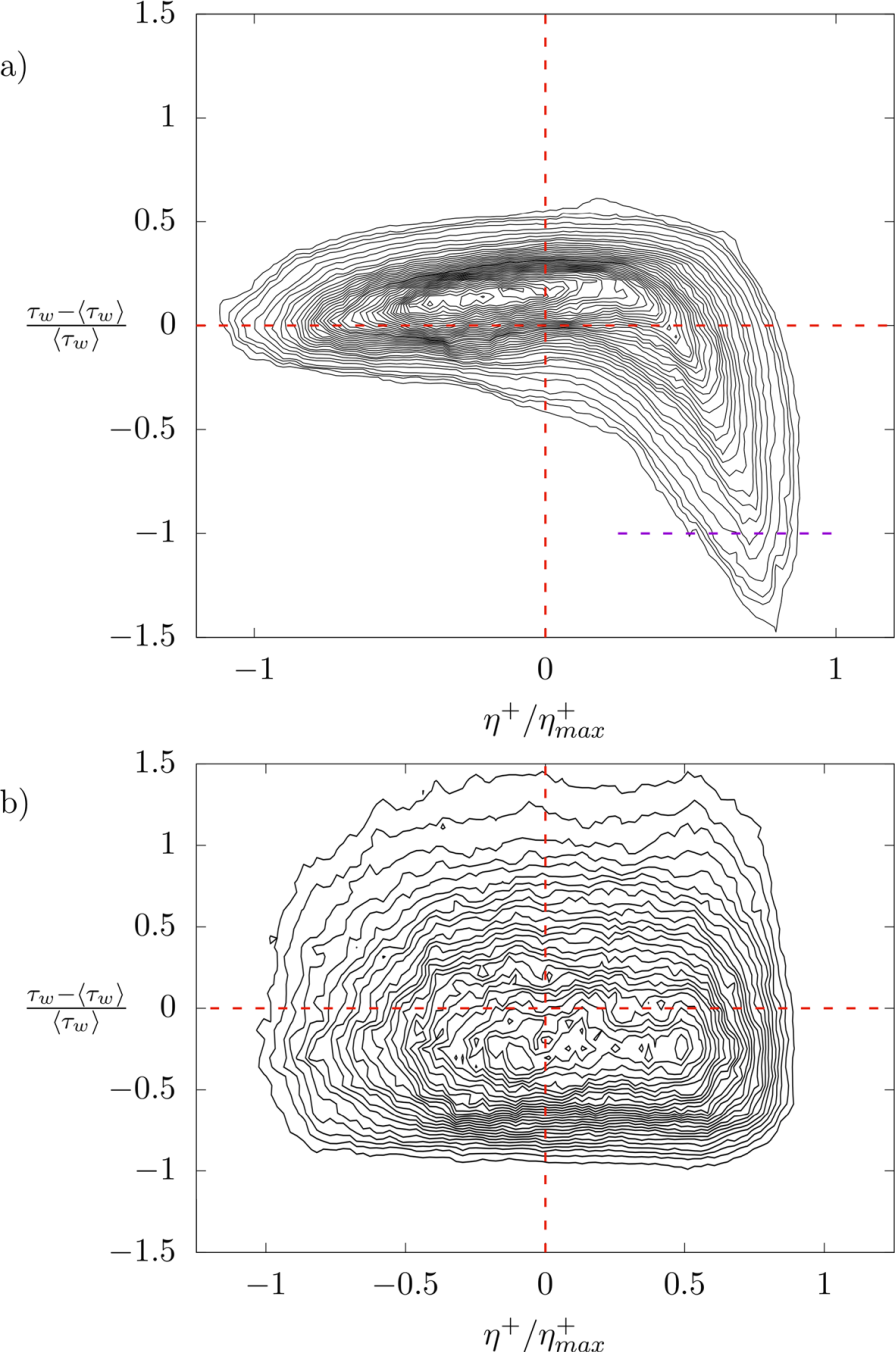
Joint Probability Density Function (PDF) between the normalized interface deformation $\eta ^{+}/\eta ^{+}_{max}$ and the corresponding shear stress deviation $\tau ^{\prime }_{w}=(\tau _{w} - \langle \tau _{w} \rangle )/\langle \tau _{w} \rangle $. **a** refers to the top wall (*z*^+^ = 200, i.e. close to the interface). **b** refers to the bottom wall (*z*^+^ = 0, i.e. far from the interface)

To quantify more closely the correlation between the interface deformation and the shear stress inversions at the wall (recirculation), we finally present a joint probability density function between the normalized interface deformation $\eta ^{+}/\eta ^{+}_{max}$ and the corresponding shear stress deviation $\tau ^{\prime }_{w}=(\tau _{w} - \langle \tau _{w} \rangle )/\langle \tau _{w} \rangle $. Results, which are shown in Fig. 8, refers to the top wall (panel a) and to the bottom wall (panel b). The most interesting situation is observed at the top wall (Fig. 8a). For negative values of $\eta ^{+}/\eta ^{+}_{max}$ (i.e. for interface throats), the interface deformation and the wall shear stress are essentially uncorrelated. The effect of the interface is in this case limited to an effective shear stress modulation that narrows the distribution shear stress distribution around $\tau ^{\prime }_{w}= 0$. The situation changes remarkably when we consider positive values of $\eta ^{+}/\eta ^{+}_{max}$ (i.e. for interface crests). In this case, we observe a strong correlation between $\eta ^{+}/\eta ^{+}_{max}$ and $\tau ^{\prime }_{w}$. In particular, when $\eta ^{+}/\eta ^{+}_{max}$ increases , then $\tau ^{\prime }_{w}$ becomes progressively larger in magnitude (but with negative sign). In particular, when $\eta ^{+}/\eta ^{+}_{max} \to 1, \tau ^{\prime }_{w}<-1$. These events represent exactly the generation of shear stress inversions with the appearance of flow recirculation regions. This plot undoubtedly demonstrates the strong correlation between the presence of wave crests and the flow recirculation close to the wall.

By contrast, at the bottom wall (far from the interface, Fig. 8b) the correlation between $\eta ^{+}/\eta ^{+}_{max} $ and $\tau ^{\prime }_{w}$ is completely different. Specifically, we observe that the joint PDF has in this case a broader area, typical of uncorrelated variables ($\tau ^{\prime }_{w}$ changes almost independently of $\eta ^{+}/\eta ^{+}_{max}$). Yet, no shear stress inversions (i.e. no $\tau ^{\prime }_{w}<-1$) are observed.

## Conclusions

In this work, the turbulent Poiseuille flow of two immiscible liquid layers inside a flat channel has been studied using Direct Numerical Simulation (DNS). We considered a mixture composed by two liquid layers with matched density but different viscosity. A configuration in which a thin layer with smaller viscosity (layer 1) moved on top of a thick layer (layer 2) with larger viscosity has been analysed. The thickness ratio between the two liquid layers was *h*_2_/*h*_1_ = 9. The simulations were run at a reference shear Reynolds number *Re*_*τ*_ = 100 and different values of the viscosity ratio, *λ* = *ν*_1_/*ν*_2_, were considered (*λ* = 1.000 − 0.875 − 0.750).

Compared to the single phase flow, the presence of a liquid-liquid interface altered significantly the overall fluid dynamics of the system. For all the values of *λ* tested, an increase of the flow rate of the thicker layer is observed. The liquid-liquid interface interacts with the near-wall turbulence structures modifying them . These modifications can be quantified considering the Probability Density Function (PDF) of the wall shear stress at the top wall, where large positive fluctuations are inhibited whereas large negative fluctuations are promoted. The increased probability of negative fluctuations determines the presence of recirculations, regions characterized by a local value of the wall shear stress *τ*_*w*_ that changes sign compared to the mane value measured at that wall. These events are strongly correlated with the interface elevation as shown by the joint probability density function of wall shear stress and interface elevation.
